# A case of transient alien hand syndrome from a very small ischemic stroke

**DOI:** 10.1002/ccr3.3166

**Published:** 2020-07-28

**Authors:** Hsin‐Chen Liu, Austin Apramian, Antonio Liu

**Affiliations:** ^1^ Department of Internal Medicine Adventist Health White Memorial Los Angeles California; ^2^ Department of Neurology Adventist Health White Memorial Los Angeles California; ^3^ Department of Neurology California Hospital Medical Center Los Angeles California

**Keywords:** alien hand syndrome, apraxia, ischemic stroke, middle cerebral artery, neurology, TIA

## Abstract

Posterior alien hand syndrome caused by small infarcts seems to be characterized by acute development and transient, yet profound, isolated motor dysfunction with a favorable prognosis unlike other cases due to larger MCA infarcts.

## Graphical abstract

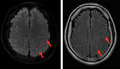

## 1 INTRODUCTION

Although not precisely defined, alien hand syndrome (AHS) has been described as a disorder of foreign limb sensation with involuntary, complex, and purposeful movements. AHS was first documented in 1908, which was described by Goldstein as “a type of apraxia with a feeling of estrangement between the patient and his hand.” In one case, a 57‐year‐old woman was unable to control her left hand from grabbing her throat. This patient's subsequent autopsy revealed an acute infarct in the right hemisphere and corpus callosum.^1^, ^2^ Since the first report, several studies have shown similar signs and symptoms of this disease in the last few decades. Previous literature has suggested that etiologies of this rare movement disorder include vascular malformation, ischemic or hemorrhagic stroke, head trauma, corticobasal degeneration, Alzheimer's disease, neurosurgery, and brain tumor.^3^, ^4^, ^5^, ^6^, ^7^, ^8^ Neurodegenerative diseases are known to be the leading cause of AHS.

In this case report, we present a young obese male patient with the chief complaint of “I thought my kids were playing a prank on me.” He was startled by a right hand that “came out of nowhere” rising up to touch his face when he was sitting on the toilet about to have a bowel movement. Symptoms lasted for at most an hour, with similar timing to a TIA‐like presentation.

## 2 CASE

A 45‐year‐old right‐handed obese man presented to emergency room with a chief complaint of being startled by his own right hand that suddenly, without his awareness, came into his right visual field approaching his face. At that moment, he was sitting on the toilet about to have a bowel movement. The bathroom door was not closed completely, so he thought his young children were playing a prank on him. Upon realizing that this “hand” was actually attached to his own body, he was shocked and found the whole situation incomprehensible. He described this “hand” as doing a wave like motion in the air. He was able to clean himself with his left hand, stood up, and called for his wife. When he arrived at ER via private vehicle, his symptoms had resolved and he had “regained control over” his right upper extremity. At that time, the patient denied any numbness, tingling, weakness, confusion, slurred speech, or any focal neurological deficits. He also denied any chest pain, shortness of breath, abdominal pain, fevers, or chills. At the time of the incident, the patient proceeded to finish his bowel movement and had no incontinence

Past Medical History—Patient obese and had chest pain 6 months prior to current admission requiring an overnight admission at another hospital in the area. A review of those medical records revealed normal EKG and troponin levels. During this past admission, the cardiologist did a left heart catheterization and determined there was no stenosis, and the left ventriculogram was normal. Conclusion for that chest pain episode was costochondritis. He denied tobacco, alcohol, or illicit drug use.

**FIGURE 1:**
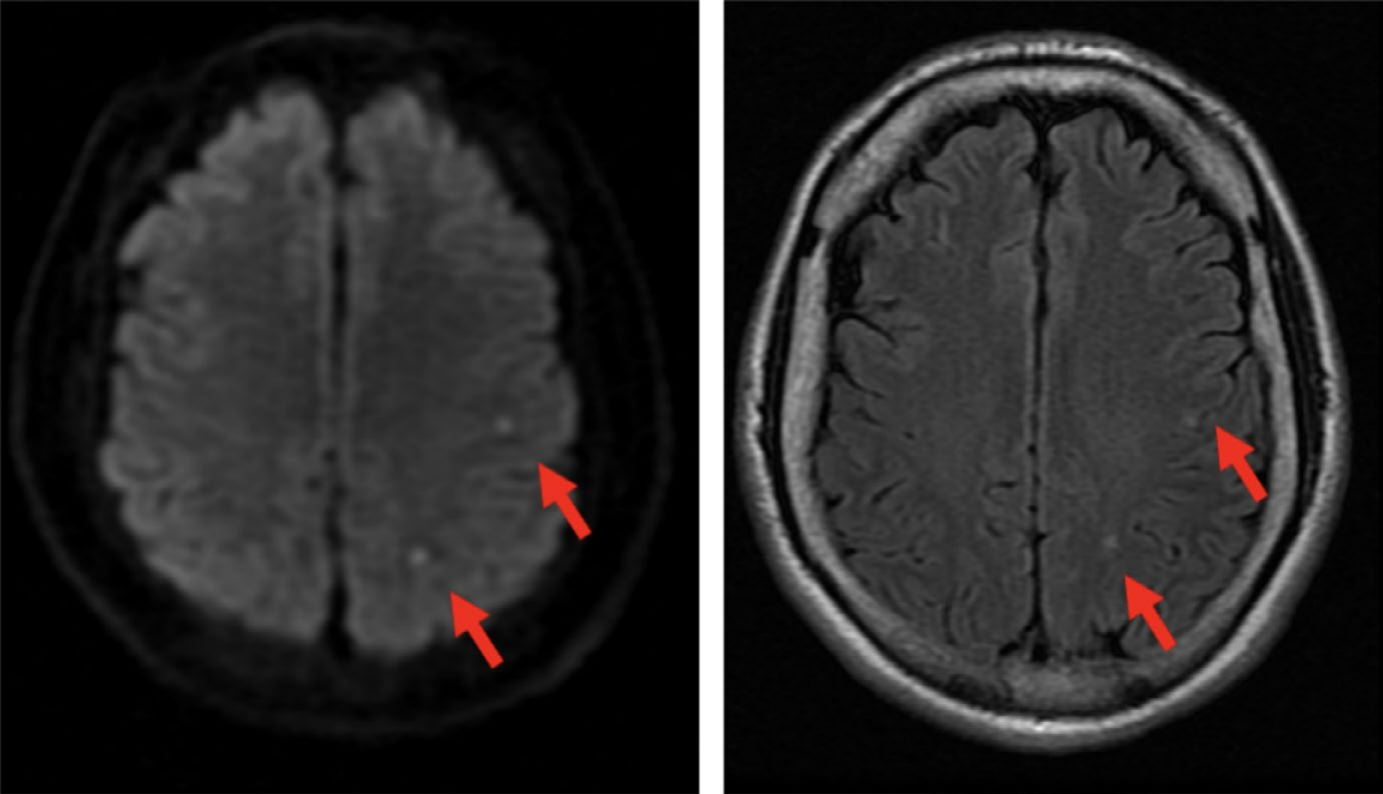
MRI DWI and Flair sequence showing two tiny infarcts within the territory of posterior left MCA

On examination, the patient had a BMI of 33. Temperature was 98.3F, and he was never febrile throughout the whole stay. His blood pressure was 108/61, heart rate was 51, and atrial fibrillation was never documented during his stay. He was alert and oriented to person, place, time, and situation. Cranial nerve examination was normal with no visual field deficit or neglect. Motor strength was 5/5 in bilateral upper and lower extremities. Tone and bulk were normal with no tremor, myoclonus, asterixis, dystonia, or chorea. Sensory tests revealed response to light touch, ice sensation, and proprioception. Asking him to hold his right upper extremity up in air with eyes closed did not result in any abnormal movement. His gait was normal. Finger to nose testing was intact with no ataxia and no dysdiadochokinesia. CT and CTA of head were both negative. MRI of head, however, showed two small embolic‐like events within the left middle cerebral artery territory within the parietal lobe on the DWI and flair sequences (Figure 1). His echocardiogram showed a 55% EF and no wall abnormality. Carotid duplex showed 0%‐15% stenosis on both internal carotid arteries. His basic chemistry, cell count and platelet, GFR, and liver function panel were all normal: INR 1.0, PTT 25.4, glucose 94, LDL 109, triglyceride 278, and HDL 36. His hypercoagulable state workup was all negative.

He remained well and was discharged home with Aspirin 81 mg, Plavix 75 mg, and Lipitor 80 mg daily. He was referred to a cardiologist for a prolonged implantable cardiac monitoring device. A follow‐up phone call 8 months after discharge revealed no recurrence, and he has returned to his usual state of health.

## 3 DISCUSSION

Although criteria have yet to be established to guide the diagnosis of AHS, Doody and Jankovic defined it as limb with foreign sensation with observable involuntary motor activity while Feinberg described it as “unwilled and uncontrollable movements of an extremity not due to a movement disorder along with the extremity's action that are experienced as involuntary and frequently contrary to the patient's intention.”^5^, ^9^

In 1992, Feinberg categorized AHS into two subtypes: frontal and callosal variants. The frontal form of anterior AHS involves the left hemisphere and dominant hand. Patients with frontal variant of AHS frequently exhibit impulsive groping, grasping, or compulsive manipulation of objects.^10^ Patients recognize that the limbs belong to them but find controlling movements of the affect limbs challenging. Lesions are primarily located in frontal lobe, affecting medial prefrontal cortex, supplementary motor area with or without involvement of corpus callosum. The most common etiology of frontal variant of AHS is acute stroke secondary to occlusion of anterior communicating artery.^2^

Unlike the frontal variant, callosal variant AHS affects exclusively the nondominant hand and presents with intermanual conflict clinically. Intermanual conflict is defined as unintended purposeful autonomous movements, where the affected limb will involuntarily undo the acts of contralateral arm.^11^ For example, a patient may open cupboard door with her right hand and her left hand would close it again involuntarily and uncontrollably.^12^ The callosal variant of anterior AHS can evolve into callosal disconnection syndrome with clinical features such as apraxia, tactile anomia, agraphia , visual anomia, neglect, and alexia in addition to intermanual conflict.^13^ Callosal variants are known to be caused by lesions in corpus callosum. They are potential complications of surgical intervention (tumor, trauma, or callosotomy for refractory epilepsy), demyelination (multiple sclerosis), and/or vascular lesions (hemorrhages from aneurysm, or ischemia).^10^

In 1998, Ay et al. presented an 81‐year‐old patient with left‐sided sensory deficit and involuntary movement secondary to subacute infarction in posterior cerebral artery territory, including right thalamus, hippocampus, inferior temporal lobes, splenium of corpus callosum, and occipital lobe.^14^ To distinguish from the anterior variants with pathology mainly from frontal lobe and corpus callosum, posterior variant was termed for its involvement in posterior cortical/subcortical areas.^15^ These patients unintentionally withdrew their affected limb from environmental contact, experienced uncoordinated hand movements, or experienced involuntary levitations.^10^ Patients may complain about arms striking their faces repetitively against their wills.

Our patient was different from other patients suffering from posterior AHS with his acute development, small affected area on imaging, isolated motor dysfunction, and TIA‐like presentation. Posterior AHS is a rare clinical condition, and its disease progression and long‐term prognosis have yet to be elucidated. Most posterior AHS are subacute from neurodegeneration such as corticobasal degeneration. Sudden onset of posterior AHS occasionally develops after acute stroke. This case is unique as our patient, with low risk factors for stroke, presents with posterior AHS‐like symptoms without sensory deficits after acute parietal embolic CVA. As the complaints of an “alien limb” often prime to the diagnosis of psychiatric illness or delirium,^8^ it is important for clinicians to approach these rare abnormal limb movements with great caution as its pathological findings and neurological damage have been well documented in prior literature.

This case report also highlights a favorable prognosis of AHS if caused by a small ischemic CVA. This patient's symptoms resolved spontaneously prior to ED arrival. Unlike previous reports of stroke‐induced AHS, MRI study of this patient only shows two small embolic lesions in the parietal lobe rather than massive infarcts in middle cerebral artery territory. Although clinical outcomes after long‐term rehabilitation are limited in data, some studies show that significant symptom relief may take up to 6 months, while some studies state no relevant improvement of impulsive behaviors after up to 12 months of follow‐up.^13^ Transient AHS has only been documented in limited studies from our literary review. Unlike most other AHS cases, this patient recovered completely without neurological deficit.

In summary, AHS is a rare neurological disorder, which is characterized by involuntary movements and foreign sensations of the affected limbs. Common etiologies include neurodegenerative disease, surgery, tumor and ischemia and hemorrhagic stroke. The underlying mechanism of AHS is still unclear even after more than 100 years since its first documentation by Goldstein. There is no established treatment for AHS. Current literature reviews suggest treating the underlying causes, such as acute stroke of TIA.^16^ In our case, a patient with stroke emboli‐induced AHS resolved spontaneously, and the patient had no neurological deficit. This patient was treated with dual‐antiplatelet and high‐dose statin therapy for secondary stroke prevention.

## CONFLICT OF INTEREST

All authors involved certify that they have NO affiliations with or involvement in any organization or entity with any financial interest (such as honoraria; educational grants; participation in speakers' bureaus; membership, employment, consultancies, stock ownership, or other equity interest; and expert testimony or patent‐licensing arrangements), or nonfinancial interest (such as personal or professional relationships, affiliations, knowledge, or beliefs) in the subject matter or materials discussed.

## AUTHOR CONTRIBUTIONS

HL: designed the study and wrote the initial draft of the manuscript. AA: contributed to analysis and interpretation of data, and prepared the final manuscript. All authors: have contributed to data collection and interpretation, and critically reviewed the manuscript. All authors: approved the final version of the manuscript and agree to be accountable for all aspects of the work in ensuring that questions related to the accuracy or integrity of any part of the work are appropriately investigated and resolved.
